# Supplementary material for: Revised cytoarchitectonic mapping of the human premotor cortex identifies seven areas and refines the localisation of frontal eye fields
Source: Commun Biol. 2025 Aug 1;8:1143. doi: 10.1038/s42003-025-08528-4 (PMC12317009; doi:10.1038/s42003-025-08528-4)
Supplement: Supplementary file 1 — Spplementary information [file 42003_2025_8528_MOESM1_ESM.pdf]

Supplementary information

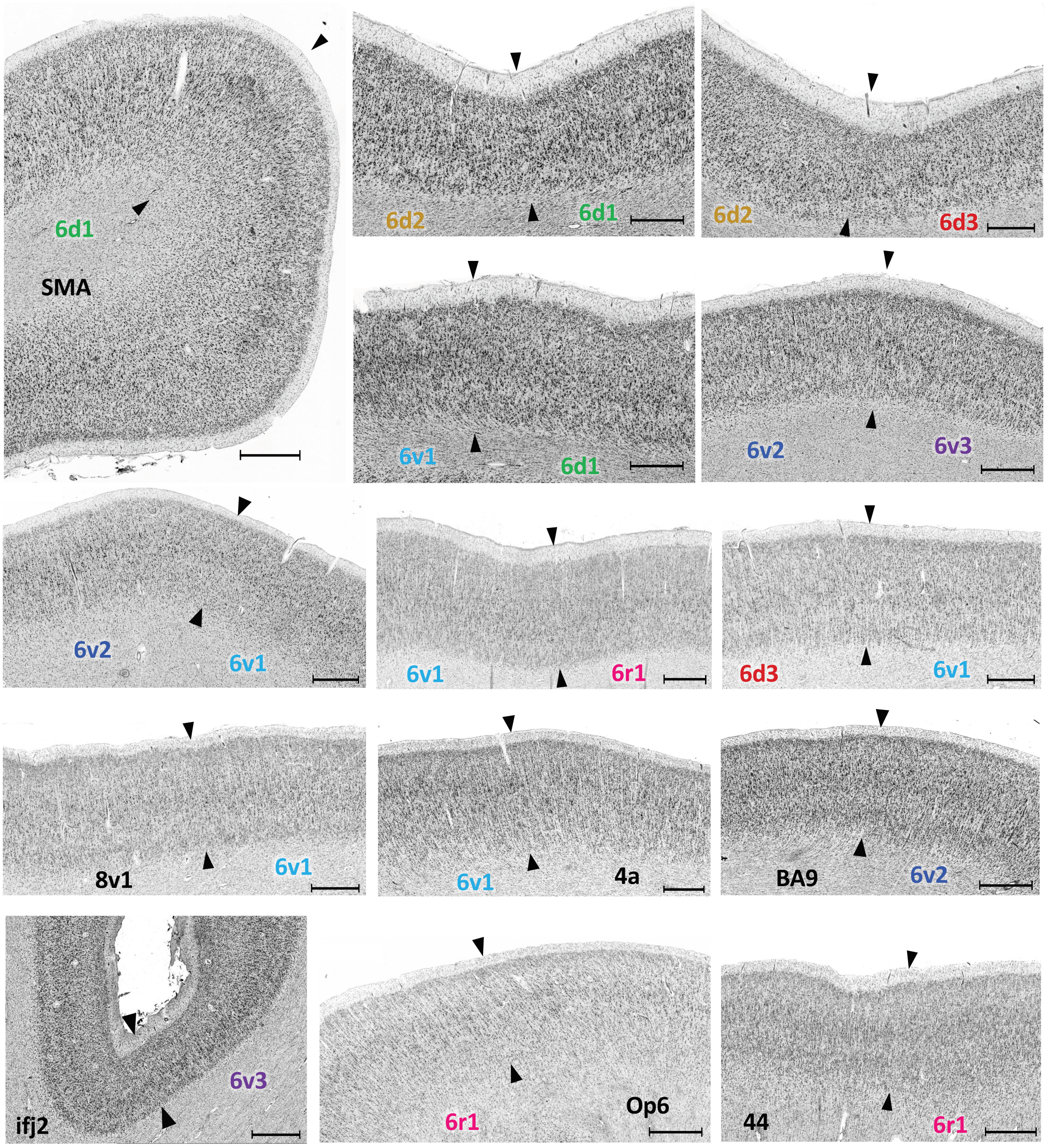

Supplementary Figure 1 Examples of cytoarchitectonic borders between the areas PM areas and to neighbouring areas. Photomicrographs of coronal, cell-body stained sections. Arrows mark the location of borders. Areas 6d1, 6d2 and SMA, preSMA differ by a less clear horizontal layering, a less sharp separation of layer IIIa/b as well as a relatively small cell size in layer IIIc in SMA, preSMA[1]. While area SMA still shows a peak in cell density in layer IIIc, in area pre-SMA layer V was particularly prominent with a high density. Area pre-SMA reveals an unsharp boundary between layers II and III, in contrast to area 6d2. Areas 8d1, 8d2, and 8v1[2] differ from 6d2, 6d3 and 6v1 by a thin layer IV (dysganular). Layer II is less sharply separated from the adjacent layers, the pyramidal cells especially in Layer IIIc are smaller and layer V is less cell dense. Brodmann Area 9 (BA9) shows in comparison to 6v1, 6v2 and 6v3 a distinct inner granular layer IV, larger pyramidal cells in layer IIIc and a lower cell density in the upper part of layer V. Areas 6v2 and 6v3 bordered area ifj2[3], a dysgranular area with a lower cell densities in layers II and V. In comparison with Area 6r1, layer IV of area 44[4] is more clearly visible and it shows a clearly visible cortical layering, larger pyramidal cells in layer III as well as a less cell dense layer V. Areas Op6[5] and 6r1 differ mainly by a more prominent layer IV and a lower cell density layer V in Op6 and more pronounced vertical columns in 6r1. Scale bars = 1 mm.

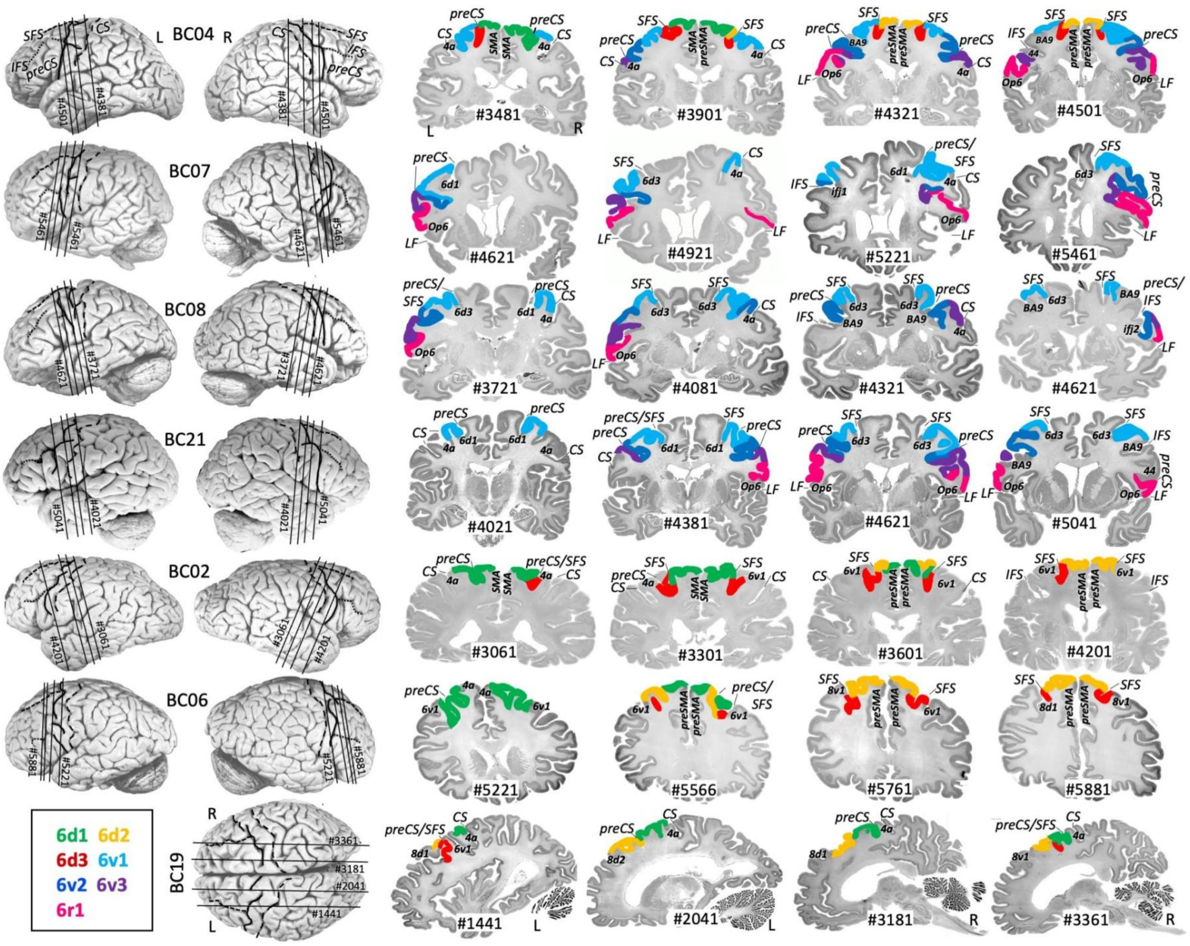

Supplementary Figure 2 Localisation of PM areas in coronal (BC02, 04, 06, 07, 08, BC21) and sagittal sections (BC19) of seven post mortem brains. Approximate locations of sections are marked by straight lines on the brain surfaces. Sulci are marked with (dotted) lines, and neighbouring areas are labelled in bold italics. In addition to the individual surface maps of six brains shown in Figure 2, this sectional views illustrate the location of areas, their borders within the depth of the sulci, as well as their interindividual variability. Area 6d3 is located within the SFS and does not extend onto the surface of neighbouring gyri. The caudally directed horizontal extension of the inferior preCS (approx. on the level of the IFS), which indicates the border between 6v2 and 6v3 has a variable depth between the hemispheres. For example, in the right hemispheres of brains BC07 and BC21, it is very deep and additionally folded, resulting in large parts of 6v2 and 6v3 being located within the sulcus. In contrast, in the right hemisphere of BC04, the sulcus shallower, and large parts of 6v2 and 6v3 are located on the gyral surface. BC, brain code; CS, central sulcus; preCS, precentral sulcus; IFS, inferior frontal sulcus; SFS, superior frontal sulcus; L, R, left and right hemisphere. For an overview of the mapped brains and areas see Table 4.

Fig.	Study	x	y	z	Location in area (p-value)
6A - FEF	Amiez et al. (2006) – Prosaccades	-38	-10	54	6v1 (0.4)
	Anderson et al. (2012) – Prosaccades in darkness	-38	-10	44	-
	Anderson et al. (2012) – Prosaccades in darkness	-26	-8	52	6d3 (0.4), 6d1 (0.3)
	Cieslik et al. (2016) - Prosaccades	-40	-2	50	6v1 (0.5), 6v2 (0.2)
	De Pasquale et al. (2010) - Prosaccade	-27	-12	53	6d1 (0.5)
	Kastner et al. (2007) - Prosaccade	-35	-12	49	-
	Koyama et al. (2004) - Prosaccade	-26	-12	54	6d1 (0.5)

	Meyyappan et al. (2020) - Spatial and feature-based task cue-evoked activity	-27	-1	52	6d3 (0.6)
	Michalka et al. (2015) - Attend visual > Attend auditory	-33	-7	47	-
	Nee and D'Esposito (2016) – temporal and contextual control	-24	4	54	6d3 (0.6)
	Paus (1996) – Ocolumotor tasks	-32	-5	50	6v1 (0.3)
	Tamber-Rosenau et al. (2018) - Prosaccades	-24	-10	46	-
	Vossel et al. (2012) - Valid trials > Baseline	-29	-6	61	-
	Wen et al. (2012) - Attend > Passive view	-30	-3	54	6v1 (0.5), 6d3 (0.22)
	Yeo et al. (2011) - Saccadic eye movements	-26	-6	48	6d3 (0.4), 6d1 (0.2)
	Amiez and Petrides (2018) - Saccadic eye movements	-40	-5	53	6v1 (0.6)
	Blanke et al. (2000) - electrical cortical stimulation	-40	-7	53	6v1 (0.4)
	Petit and Haxby (1999) - pursuit eye movements	-35	-21	50	6v1 (0.5)
	Grosbras et al. (2005)- saccadic eye movements	-30	-11	55	6d1 (0.3)
	Astafiev et al. (2003) - attending, looking, and pointing to a peripheral visual location	-29	-14	55	6d1 (0.5)
	Corbetta et al. (1998) - attentional and saccadic shifts	-27	-13	46	-
	Gagnon et al. (2002) - saccades	-30	-12	57	6d1 (0.3)
	Gitelman et al. (2002) - Visuomotor search	-33	-9	52	6v1 (0.2)
	Luna et al. (1998) - visually guided saccades	-30	-7	53	-
	Heinen et al. (2006) - ocular baseball, control eye-movement task	-34	-15	55	6d1 (0.2)
	Petit and Beauchamp (2003) - eye, visually guided head and gaze movements	-26	-18	58	6d1 (0.6)
	Beauchamp et al. (2001) - overt and covert attentional shifts	-29	-9	53	6d1 (0.3)
	Heide et al. (2001) – memory-guided sequences of saccadic eye movements	-28	-12	52	6d1 (0.4)
	Petit et al. (2009) - large and small visually guided saccades	-28	-4	52	6d3 (0.3)
	Petit et al. (2015) – visually guided saccadic eye movements	-26	-4	52	6d3 (0.6), 6d1 (0.2)
6B - iFEF	Kato and Miyauchi (2003) - intentional eyeblink tasks	-52	-3	43	6v2 (0.3), 6v3 (0.3)
	Luna et al. (1998) - visually guided saccades	-44	6	44	6v2 (0.8)
	Petit and Beauchamp (2003) - eye, visually guided head and gaze movements	-45	-14	40	4p (0.5), 3b (0.4)
	Beauchamp et al. (2001) - overt and covert attentional shifts	-42	-3	37	6v2 (0.4)
	Heide et al. (2001) – memory-guided sequences of saccadic eye movements	-45	-10	47	4a (0.3)
	Petit et al. (2009) - large and small visually guided saccades	-49	-2	42	6v2 (0.5)
	Petit et al. (2015) – visually guided saccadic eye movements	-44	-6	50	-
	Derrfuss et al. (2012) - saccades vs. button presses	-55	-3	42	6v3 (0.3)
	Amiez et al. (2009)- saccadic eye movements	-52	-4	44	6v2 (0.4), 6v3 (0.2)
6C - motion	Lafleur et al. (2002) - foot	-28	-5	63	6d2 (0.2)
	Fink et al. (1997) - leg	-30	-14	66	6d1 (0.4)
	Chen et al. (2018) – arm/reaching	-22	-10	62	6d1 (0.5)
	Gertz et al. (2015) – arm/reaching	-26	-6	64	6d2 (0.3), 6d1 (0.3)
	Bernier et al. (2012) – arm/reaching	-27	-12	60	6d1 (0.3)
	Cavina-Pratesi et al. (2018) – arm/reaching	-17	-23	68	-
	Fabbri et al. (2010) – arm/reaching	-31	-13	62	-
	Kuhtz-Buschbeck et al. (2001) – hand/grasping	-58	4	38	6v3 (0.4)
	Jacobs et al. (2010) – hand/grasping	-56	2	36	6v3 (0.5)

	Keisker et al. (2009) – hand/grasping	-53	3	33	6v3 (0.6)
	Keisker et al. (2010) – hand/grasping	-44	-2	32	6v3 (0.3)
	Schubotz et al. (2010) - mouth	-52	-10	38	-
	Kern et al. (2019) - mouth	-62	7	32	6v3 (0.3)
6D - perception	Schubotz et al. (2001), visual, spatial	-23	-14	56	6d1 (0.5)
	Schubotz et al. (2001), visual , object	-41	4	37	6v2 (0.8), ifj2 (0.2)
	Schubotz et al. (2001), visual, temporal	-46	11	6	44 (0.3), Op6, Op8 (0.2),
	Schubotz et al. (2001), visual	-45	3	28	6v3 (0.5)
	Schubotz et al. (2003), auditory, spatial	-21	-11	57	6d1 (0.4)
	Schubotz et al. (2003), auditory, object	-44	4	34	6v2 (0.4), 6v3 (0.4)
	Schubotz et al. (2003), auditory, temporal	-46	1	6	Op6 (0.7)
	Schubotz et al. (2003), auditory	-53	2	5	Op6 (0.8)

Supplementary Table 1. Location of coordinates visualised in Figure 6 in the maps of Julich-Brain areas (in MNI Colin27). The p-value indicates the probability of the respective area at the position of the coordinate point.

Area in Glasser's map	3D correlation with PM area (correlation coefficient)
6ma L	6d2 (0.56), 6d1 (0.10)
6ma R	6d2 (0.41), 6d3 (0.12)
6mp L	6d1 (0.39)
6mp R	6d1 (0.51)
6a L	6d3 (0.43), 6d1 (0.15)
6a R	6d3 (0.53), 6v1 (0.13)
6d L	6d1 (0.21), 6v1 (0.16)
6d R	6v1 (0.17)
55b L	6v2 (0.34)
55b R	6v2 (0.32), 6v3 (0.05)
6v L	6v3 (0.41)
6v R	6v3 (0.55), 6r1 (0.11), 6v2 (0.10)
6r L	6r1 (0.39)
6r R	6r1 (0.33)

Supplementary Table 2 3D correlation of left and right hemispheric areas 6ma, 6mp, 6a, 6d, 55b, 6v and 6r of the multimodal map by Glasser et al. [6] and PM areas in ICBM152 space. 6ma, anterior supplementary motor area; 6mp, posterior supplementary motor area; L, left hemisphere, R, right hemisphere

	BC11	BC06	BC02	BC04	BC10	BC19	BC05	BC20	BC01	BC09
**6d1 L**	4219	5661	4420	2159	3242	5689	3930	5261	2228	3128
**6d1 R**	3998	6180	4322	2882	2984	4376	4193	6546	1179	4161
**6d2 L**	4619	2724	2324	1238	3546	3479	1543	3933	2899	2809
**6d2 R**	4876	1955	3128	2198	3033	3452	2125	3812	2998	3498
**6d3 L**	3112	937	2968	3068	2001	2755	1452	1501	2021	664
**6d3 R**	3576	922	2824	2222	1967	1667	1355	1304	2141	1054
	**BC11**	**BC07**	**BC04**	**BC10**	**BC05**	**BC20**	**BC21**	**BC01**	**BC08**	**BC09**

**6v1 L**	6166	4463	5389	2164	1993	5047	5145	4324	5131	2232
**6v1 R**	5490	4107	4544	3251	2125	4444	5913	5767	5711	4232
**6v2 L**	3334	1961	2526	898	2215	2705	3517	771	2245	2117
**6v2 R**	3869	2829	1889	2430	921	617	2165	2001	2440	2412
**6v3 L**	1995	1972	1397	733	1369	1046	2230	2345	2398	1713
**6v3 R**	2230	1880	3215	699	1687	3031	2471	943	762	784
**6r1 L**	2726	2209	2121	2200	1145	1687	1933	438	1155	1328
**6r1 R**	2659	3300	1384	2062	1404	2073	2567	463	530	1396

Supplementary Table 3 Volumes (in mm^3) of areas 6d1, 6d2, 6d3, 6v1, 6v2, 6v3 and 6r1 in the left (L) and right (R) hemisphere in the individual brains (BC, Brain code) after shrinkage correction.

References

1 Ruan, J. *et al.* Cytoarchitecture, probability maps, and functions of the human supplementary and pre-supplementary motor areas. *Brain Struct Funct* **223**, 4169-4186 (2018). https://doi.org/10.1007/s00429-018-1738-6

2 Amunts, K., Mohlberg, H., Bludau, S. & Zilles, K. Julich-Brain: A 3D probabilistic atlas of the human brain's cytoarchitecture. *Science* **369**, 988-992 (2020). https://doi.org/10.1126/science.abb4588

3 Ruland, S. H. *et al.* The inferior frontal sulcus: Cortical segregation, molecular architecture and function. *Cortex* **153**, 235-256 (2022). https://doi.org/10.1016/j.cortex.2022.03.019

4 Amunts, K. *et al.* Broca's region revisited: cytoarchitecture and intersubject variability. *J Comp Neurol* **412**, 319-341 (1999). https://doi.org/10.1002/(sici)1096-9861(19990920)412:2<319::aid-cne10>3.0.co;2-7

5 Unger, N. *et al.* Cytoarchitectonic mapping of the human frontal operculum-New correlates for a variety of brain functions. *Front Hum Neurosci* **17**, 1087026 (2023). https://doi.org/10.3389/fnhum.2023.1087026

6 Glasser, M. F. *et al.* A multi-modal parcellation of human cerebral cortex. *Nature* **536**, 171-178 (2016). https://doi.org/10.1038/nature18933
